# Risk factors for surgery-related muscle quantity and muscle quality loss and their impact on outcome

**DOI:** 10.1186/s40001-021-00507-9

**Published:** 2021-04-23

**Authors:** Laura van Wijk, Stijn van Duinhoven, Mike S. L. Liem, Donald E. Bouman, Alain R. Viddeleer, Joost M. Klaase

**Affiliations:** 1 Department of Hepatobiliary Surgery and Liver Transplantation, University Medical Center Groningen, Hanzeplein 1, PO Box 30001, 9700 RB Groningen, the Netherlands; 2 Department of Surgery, Medisch Spectrum Twente, Koningsplein 1, 7512 KZ Enschede, the Netherlands; 3 Department of Radiology, Medisch Spectrum Twente, Koningsplein 1, 7512 KZ Enschede, the Netherlands; 4 Department of Radiology, University Medical Center Groningen, Hanzeplein 1, PO Box 30001, 9700 RB Groningen, the Netherlands

**Keywords:** Surgery-related muscle quantity loss, Surgery-related muscle quality loss, Psoas muscle index, Total psoas area, Liver resection, Colorectal liver metastasis

## Abstract

**Background:**

Surgery-related loss of muscle quantity negatively affects postoperative outcomes. However, changes of muscle quality have not been fully investigated. A perioperative intervention targeting identified risk factors could improve postoperative outcome. This study investigated risk factors for surgery-related loss of muscle quantity and quality and outcomes after liver resection for colorectal liver metastasis (CRLM).

**Methods:**

Data of patients diagnosed with CRLM who underwent liver resection between 2006 and 2016 were analysed. Muscle quantity (psoas muscle index [PMI]), and muscle quality, (average muscle radiation attenuation [AMA] of the psoas), were measured using computed tomography. Changes in PMI and AMA of psoas after surgery were assessed.

**Results:**

A total of 128 patients were analysed; 67 (52%) had surgery-related loss of muscle quantity and 83 (65%) muscle quality loss. Chronic obstructive pulmonary disease (COPD) (*P* = 0.045) and diabetes (*P* = 0.003) were risk factors for surgery-related loss of muscle quantity. A higher age (*P* = 0.002), open resection (*P* = 0.003) and longer operation time (*P* = 0.033) were associated with muscle quality loss. Overall survival was lower in patients with both muscle quantity and quality loss compared to other categories (*P* = 0.049). The rate of postoperative complications was significantly higher in the group with surgery-related loss of muscle quality.

**Conclusions:**

Risk factors for surgery-related muscle loss were identified. Overall survival was lowest in patients with both muscle quantity and quality loss. Complication rate was higher in patients with surgery-related loss of muscle quality.

## Introduction

Resection with curative intent is the treatment of choice for colorectal liver metastasis (CLRM) [1, 2]. Despite advances in surgical techniques and perioperative care, liver resection still causes substantial rates of postoperative morbidity and mortality [3–5]. Postoperative morbidity may result in prolonged hospital stays, increased healthcare costs, and potentially decreased long-term survival [6, 7]. In recent years, awareness has grown of using body composition variables as predictors for postoperative outcomes in surgery. Studies have demonstrated that low muscle mass referred to as sarcopenia negatively affects postoperative outcomes after resection for CRLM [8, 9], and preoperative sarcopenia is associated with poor overall survival in patients with various solid tumours [10]. In addition, there is increasing evidence that preoperative low muscle radiation attenuation as a measure of muscle quality, also referred as myosteatosis, is also an important prognostic factor for impaired outcome in patients with cancer [11–13].

Although the impact of preoperative body composition variables has been well described in literature, less studies have investigated the process of surgery-related changes in muscle quantity and quality. Recent reports have suggested that loss of muscle quantity after surgery is associated with decreased quality of life and short-term outcomes [14–16]. In addition, the negative impact of this so-called surgery-related muscle loss (SML) on long-term survival after pancreatic surgery was recently demonstrated [17]. However, these studies only concern muscle quantity. There is minimal literature describing surgery-related changes in muscle quality [18].

Furthermore, through identifying risk factors for surgery-related loss of muscle quantity and quality, perioperative intervention might prevent or reduce SML and subsequently improve postoperative outcomes. Being aged above 65 years and diabetes were reported to be independent risk factors for clinically relevant loss of muscle quantity within 1 week of gastric cancer surgery [16]. Risk factors for surgery-related loss of muscle quality have not yet been described. The aim of this study is to identify risk factors for surgery-related loss of both muscle quantity and quality after liver resection for CRLM. In addition, we also investigate the impact of surgery-related loss of muscle quantity and quality on overall survival.

## Material and methods

### Patients

This retrospective study included patients who had undergone liver resection for CRLM at Medisch Spectrum Twente, Enschede, The Netherlands between October 2006 and September 2016. Patients were selected from a liver resection database containing prospectively collected patient, treatment, and outcome data. The inclusion criteria were (i) patients resected for CRLM with available and (ii) pre- and postoperative abdominal computed tomography (CT) scans (within 6 weeks before and 3 weeks after surgery). All patients were treated according to a multimodal Enhanced Recovery After Surgery (ERAS) pathway [19]. Postoperative CT scans were performed as part of a standard protocol and used as the baseline for oncological follow-up from 2011 onward. This study was approved by the Institutional Review Boards of the University Medical Center Groningen (Research registration number: 201800063) and Medisch Spectrum Twente.

### Data collection

For each patient enrolled in the study, the following data were collected: patient characteristics, including age, sex, patient length, preoperative body mass index (BMI), preoperative carcinoembryonic antigen (CEA) blood level, and comorbidity, including the Charlson Comorbidity Index (CCI) and American Society of Anaesthesiologists (ASA) risk score; and surgical parameters, such as the type of operation (i.e., minor [< 3 segments] or major [≥ 3 segments] resection, open or laparoscopic resection, and whether resection was combined with radiofrequency ablation [RFA]), operation time, and blood loss. Postoperative characteristics were also collected, which included all complications, complications clustered according to Clavien–Dindo scores (with major complications being defined as grade ≥ 3), and hospital length of stay. Follow-up survival data were collected from the patient charts.

### Image acquisition

When multiple CT examinations were available within 6 weeks before and 3 weeks after surgery, the CT scans closest to the day of surgery were selected. All acquired scans had a slice thickness of 1–5 mm, using a 512 × 512 matrix. After the CT images were anonymised, they were exported from the Picture Archiving and Communication System (PACS) and stored in Digital Imaging and Communications in Medicine (DICOM) format for analysis.

### Image analysis

As in previous studies investigating surgery-related muscle loss, the surgery-related change in muscles was evaluated using the Total Psoas Area (TPA) measured by abdominal CT at the level of the third lumbar vertebra [14, 15, 18]. The border of the psoas muscle was manually outlined by an experienced board (board certified radiologist, 12 years of experience, and experienced researcher) using in-house developed analysis software (SarcoMeas 0.54). The TPA was computed as the sum of all muscle voxels within the drawn cross-sectional areas of the right and left psoas muscles, where muscle is defined as a radiation attenuation from − 29 to + 150 Hounsfield units [20]. The TPA was normalised for the patient’s height by dividing the muscle area (in cm^2^) by the square of the patient’s height (in meters), resulting in the Psoas Muscle Index (PMI cm^2^/m^2^) [21]. The average muscle radiation attenuation (AMA) in Hounsfield units (HU) of the measured psoas voxels was also calculated.

### Statistical analysis

Continuous data are presented as the mean ± standard deviation or as medians and ranges as appropriate. Categorical data were presented as quantity and proportion. Descriptive statistics were used to analyse the baseline characteristics of the study population. Characteristics and variables between patients with skeletal muscle loss and those without were compared using a two-sample independent *t* test for normally distributed numerical variables; a Mann–Whitney *U* test for numerical variables that were not normally distributed; and a Pearson *X*^2^ test for binary variables. Any variable with *P* < 0.10 in the univariate analysis was included in the multivariate regression analysis. A backward multivariate regression selection analysis was performed to identify independent risk factors for surgery-related muscle quantity and quality loss. The overall survival rates after surgical resection for CRLM were determined using the Kaplan–Meier method, and differences between groups were compared using the log-rank test. The data were analysed using SPSS version 25 (IBM, Armonk, NY, USA).

## Results

### Study population

A total of 340 patients underwent surgery for CRLM during the study period. No suitable CT scans were available within the selected time frames in 155 patients and patient’s height data were unavailable for 12 patients. In 45 patients, one or both CT scans were of insufficient quality to determine the TPA (for example, because of an incompletely visualised psoas muscle or excessive noise in non-diagnostic low-dose CTs). The remaining 128 patients were included in this study’s analysis.

### Baseline and intra-operative characteristics

Table 1 lists the baseline and intra-operative characteristics of the study patients (*n* = 128). Their mean age was 65.5 ± 8.7 years; 89 (69.5%) were men; and their mean BMI was 25.6 ± 3.1 (kg/m^2^). Most patients had a Charlson Comorbidity Index (CCI) of 0 (80.5%), 19 (14.8%) patients had a CCI of I, and only six patients had a CCI of II or III (4.7%). Preoperative CT scans were performed with a mean time before operation of 28.2 ± 9.6 days, whereas postsurgical CT scans were performed after a mean of 7.1 ± 3 days. Only 15 patients (11.7%) underwent laparoscopic liver resection, which were all minor resections. Most (81.3%) of operations were resections without radiofrequency ablation. Mean operation time for all patients was 134.2 ± 61.6 min.

**Table 1:** Baseline and intra-operative characteristics in patients with and without surgery-related loss of muscle quantity or quality

Factors	Total *n* = 128 (100%)	Muscle quantity loss *n* = 67 (52%)	No muscle quantity loss *n* = 61 (48%)	*P*-value	Muscle quality loss *n* = 83 (65%)	No muscle quality loss *n* = 45 (35%)	*P*-value
Age, mean (SD), years	65.5 (8.7)	65.4 (7.7)	65.6 (9.6)	0.896	66.9 (7.8)	62.7 (9.6)	**0.008**
Sex, male	89 (69.5%)	50 (74.6%)	39 (63.9%)	0.249	62 (74.7%)	27 (60.0%)	0.085
BMI, mean (SD), kg/m2	25.6 (3.1)	25.9 (3.0)	25.5 (3.2)	0.473	25.8 (3.1)	25.5 (3.1)	0.677
CEA^a^, median (IQR), μg/L	6.3 (2.8–22.3)	5.7 (2.4–19.0)	8.0 (3.2–33)	0.146	6.5 (2.9–35.0)	5.6 (2.6–15.0)	0.322
ASA score							
I	14 (10.9%)	7 (10.4%)	7 (11.5%)	0.580	6 (7.2%)	8 (17.8%)	0.184
II	107 (83.6%)	55 (82.1%)	52 (85.2%)		72 (86.7%)	35 (77.8%)	
III	7 (5.5%)	5 (7.5%)	2 (3.3%)		5 (6.0%)	2 (4.4%)	
Comorbidities							
Diabetes	7 (5.5%)	5 (7.5%)	2 (3.3%)	0.298	5 (6%)	2 (4.4%)	0.707
Chronic obstructive pulmonary disease	6 (4.7%)	5 (7.5%)	1 (1.6%)	0.120	4 (4.8%)	2 (4.4%)	0.924
Charlson Comorbidity Index							
0	103 (80.5%)	53 (79.1%)	50 (82%)	0.786	66 (79.5%)	37 (82.2%)	0.867
I	19 (14.8%)	10 (14.9%)	9 (14.8%)		13 (15.7%)	6 (13.3%)	
II	5 (3.9%)	3 (4.5%)	2 (3.2%)		3 (3.6%)	2 (4.4%)	
III	1 (0.8%)	1 (1.5%)	0 (0.0%)		1 (1.2%)	0 (0.0%)	
CT scan							
Time-frame pre-scan and OK, mean (SD), days	28.2 (9.6)	28.4 (9.9)	27.9 (9.2)	0.755	27.1 (9.5)	30.1 (9.5)	0.091
Time-frame OK and post-scan, mean (SD), days	7.1 (3.0)	7.4 (3.4)	6.6 (2.5)	0.130	6.7 (2.7)	7.6 (3.5)	0.175
Operation type							
Laparoscopic/open	15 (11,7%)/113 (88,3%)	6 (9.0%)/61 (91%)	9 (14.8%)/52 (85.2%)	0.308	4 (4,8%)/79 (95.2%)	11 (24,4%)/34 (75,6%)	**0.001**
Minor/major	74 (57.8%)/54 (42.2%)	37 (55.2%)/30 (44.8%)	37 (60.7%)/24 (39.3%)	0.534	47 (56.6%)/36 (43.3%)	27 (60.0%)/18 (40%)	0.712
Resection/resection + RFA	104 (81.3%)/24 (18.8%)	49 (73.1%)/18 (26.9%)	55 (90.2%)/6 (9.8%)	**0.014**	65 (78.3%)/18 (21.7%)	39 (86.7%)/6 (13.3%)	0.248
Operation parameters							
Blood loss, median (IQR), mL	400 (200–750)	400 (187–700)	400 (200–800)	0.728	500 (200–763)	300 (100–625)	0.062
Operation time, mean (SD), min	134.2 (61.6)	130.2 (58.9)	138.8 (64.6)	0.434	142.7 (65)	118.1 (51.1)	**0.035**

*SD* standard deviation, *IQR* interquartile range, *BMI* body mass index, *CEA* carcinoembryonic antigen, *ASA* American Society of Anaesthesiologists, *CT* computer tomography, *RFA* radiofrequency ablation

The values given are numbers of patients unless indicated otherwise. Bold variables were considered statistically significant (*P* < 0.05)

^a^28 patients missing

### Pre- and postoperative CT measures.

The mean preoperative PMI was 6.1 ± 1.7 cm^2^/m^2^. The mean postoperative PMI was 6.0 ± 1.6 cm^2^/m^2^. In 67 (52%) of the 128 patients we found surgery-related loss of muscle quantity (PMI) with a mean loss of 7.1% ± 5.7%. Patients with loss of muscle quantity had a significantly higher preoperative PMI (6.4 [SD 1.7]) compared to patients without loss of muscle quantity (5.7 [SD 1.5]) (*P* = 0.014*)*.

The mean preoperative AMA of psoas was 45.4 ± 7.5 HU. The mean postoperative AMA of psoas was 42.5 ± 9.5 HU. In 83 (65%) patients we found surgery-related loss of muscle quality (AMA of psoas) with a mean loss of 8.1 ± 5.6 HU. The preoperative AMA of the psoas was higher (46.9 HU [7.2]) in patients with surgery-related loss of muscle quality than in patients without surgery-related loss of muscle quality (42.7 HU [SD 7.1]) *P* = 0.002). Of the patients with loss of muscle quantity (*n* = 67), most patients (*n* = 48 [72%]) had also quality loss. Of the patients with loss of muscle quality (*n* = 83), most patients (*n* = 48 [58%]) had also quantity loss. However, no significant association was found (*P* = 0.091). In this study, in the group with surgery-related loss of muscle quantity, a mean decrease in muscle volume of 7.1% was observed. In addition, in the group with quality loss, a decrease of 8.1 HU in radio density was observed.

### Comparison of pre- and intraoperative characteristics of patients with and without loss of muscle quantity

In Table 1, comparisons of baseline and intra-operative characteristics between patients with and without loss of muscle quantity are presented. No significant differences in baseline factors between the two groups were found. Examining the intraoperative characteristics, a significant difference (*P* = 0.014) was found between the percentage of combined procedures (resection and radio frequency ablation) in the group with loss of muscle quantity (26.9%) versus the group without loss of muscle quantity (9.8%). Other intraoperative characteristics, such as open versus laparoscopic, minor versus major surgery, blood loss, and operation time, were not significantly different between the two groups.

### Comparison of pre- and intraoperative characteristics of patients with and without loss of muscle quality

Patients with surgery-related loss of muscle quality were on average older (66.9 years [SD 7.8]) than patients without surgery-related loss of muscle quality (62.7 years [SD 9.6]) (*P* = 0.008). No other significant differences in preoperative factors were found (Table 1). In patients with surgery-related loss of muscle quality, the rate of laparoscopic procedures was significantly lower (4.8%) compared to the other group (24.4%) (*P* = 0.001). The duration of the operation was significant longer in the patients with surgery-related loss of muscle quality (*P* = 0.035).

### Postoperative characteristics

Overall, 52 (40.6%) patients had a complicated postoperative course. Of all types of complication, cardiopulmonary complications occurred the most, (*n* = 23 18.0%). There were no significant differences found between patients with or without surgery-related loss of muscle quantity for all types of postoperative complications and length of hospital stay. Patients with surgery-related loss of muscle quality had significantly more often a complicated postoperative course (*P* = 0.047) compared to patients without loss of muscle quality, as presented in Table 2.

**Table 2:** Postoperative characteristics of patients with and without surgery-related loss of muscle quantity or quality

	Total *n* = 128 (100%)	Muscle quantity loss *n* = 67 (52%)	No muscle quantity loss *n* = 61 (48%)	*P*-value	Muscle quality loss n = 83 (65%)	No muscle quality loss *n* = 45 (35%)	*P*-value
Complicated postoperative course	52 (40.6%)	25 (37.3%)	27 (44.3%)	0.424	39 (47%)	13 (28,9%)	**0.047**
Cardiopulmonary	23 (18.0%)	10 (14.9%)	13 (21.3%)	0.347	17 (20.5%)	6 (13.3%)	0.315
Incisional SSI	8 (6.3%)	5 (7.5%)	3 (4.9%)	0.552	6 (7.2%)	2 (4.4%)	0.534
Intra-abdominal SSI	10 (7.8%)	5 (7.5%)	5 (8.2%)	0.877	8 (9.6%)	2 (4.4%)	0.296
Infectious, other	6 (4.7%)	5 (7.5%)	1 (1.6%)	0.120	1 (1.2%)	1 (2.2%)	0.658
Bacteraemia	2 (1.6%)	2 (3.0%)	0 (0.0%)	0.174	6 (7.2%)	0 (0.0%)	0.065
Bile leak (≥ ISGLS Grade *B*)	5 (3.9%)	1 (1.5%)	4 (6.6%)	0.140	3 (3.6%)	2 (4.4%)	0.817
DGE (grade *A*, *B* or *C*)	5 (3.9%)	2 (3.0%)	3 (4.9%)	0.573	4 (4.8%)	1 (2.2%)	0.469
Thromboembolic event	3 (2.3%)	1 (1.5%)	2 (3.3%)	0.505	2 (2.4%)	1 (2.2%)	0.947
Bleeding	1 (0.8%)	1 (1.5%)	0 (0.0%)	0.338	1 (1.2%)	0 (0.0%)	0.460
Single organ failure	3 (2.3%)	1 (1.5%)	2 (3.3%)	0.505	2 (2.4%)	1 (2.2%)	0.947
Other	11 (8.6%)	5 (7.5%)	6 (9.8%)	0.632	9 (10.8%)	2 (4.4%)	0.217
Clavien–Dindo ≥ 3	18 (14.1%)	9 (13.4%)	9 (14.8%)	0.830	13 (15.7%)	5 (11.1%)	0.479
Length of hospital stay, mean (SD)	9.7 (5.3)	9.4 (4.5)	10.0 (6.2)	0.522	10.3 (4.3)	8.6 (6.8)	0.084

*SSI* surgical site infection, *ISGLS* International Study Group of Liver Surgery, *DGE* delayed gastric emptying, *SD* standard deviation, *CT* computed tomography

The values given are numbers of patients unless indicated otherwise. Bold variables were considered statistically significant (*P* < 0.05)

### Pre- and intraoperative risk factors for surgery-related loss of muscle quantity

Table 3 evaluates multiple factors that might be associated with surgery-related loss of muscle quantity. A univariate linear regression analysis suggests that male gender, the presence of chronic obstructive pulmonary disease (COPD) or diabetes are associated with higher percentages of surgery-related loss of muscle quantity (*P* < 0.10). After a backward multivariate linear regression selection analysis, the presence of COPD or diabetes were identified as independent factors associated with higher percentage amounts of surgery-related loss of muscle quantity (*P* < 0.05).

**Table 3:** Linear regression analysis of pre- and intraoperative factors associated with surgery-related loss of muscle quantity

Factors	Univariate analysis		Multivariate analysis	
	B (95% CI)	*P*-value	B (95% CI)	*P*-value
Age (per 1 year increase)	0.036 (− 0.161–0.234)	0.716		
Gender (male)	− 3.240 (− 6.928–0.448)	0.085*		
BMI (per kg/m^2^)	0.132 (− 1.063–1.326)	0.828		
CEA^a^ (per μg/L)	− 0.007 (− 0.029–0.016)	0.553		
ASA grade				
I				
II	− 2.099 (− 6.815–2.618)	0.380		
III	5.417 (− 2.078–12.911)	0.155		
Comorbidity				
Diabetes	10.778 (3.466–18.091)	**0.004**	11.173 (3.937–18.408)	**0.003**
Chronic obstructive pulmonary disease	7.307 (− 0.717–15.331)	0.074*	7.948 (0.165–15.731)	**0.045**
Surgery				
Open resection (versus laparoscopic)	1.314 (− 4.022–6.649)	0.627		
Major (versus minor)	− 1.465 (− 4.934–2.003)	0.405		
Resection + RFA ( versus only resection)	2.419 (− 1.962–6.799)	0.277		
Blood loss (per ml)	− 0.001 (− 0.003–0.001)	0.383		
Operation time (per min)	− 0.002 (− 0.026–0.030)	0.884		

*BMI* body mass index, *CEA* carcinoembryonic antigen, *ASA* American Society of Anesthesiologists, *RFA* radiofrequency ablation

Bold variables were considered statistically significant (*P* < 0.05). *Variables that were taken into the multivariable analysis (*P* < 0.010)

^a^28 patients missing

### Pre- and intraoperative risk factors for surgery-related loss of muscle quality

Table 4 evaluates multiple factors that might be associated with surgery-related loss of muscle quality. Univariate logistic regression analysis showed that a higher age, male gender, open resection and a longer operation time were associated with surgery-related loss of muscle quality (*P* < 0.10). Multivariate logistic regression analysis demonstrated that a higher age, open resection and a longer operation time were significantly associated with surgery-related loss of muscle quality (*P* < 0.05).

**Table 4:** Logistic regression analysis of pre- and intraoperative factors associated with surgery-related loss of muscle quality

Factors	Univariate analysis		Multivariate analysis	
	OR (95% CI)	*P*-value	OR (95% CI)	*P*-value
Age (per 1 year)	1.059 (1.013–1.107)	0.011*	1.082 (1.029–1.138)	**0.002**
Gender (male)	0.508 (0.234–1.103)	0.087*		
BMI (per kg/m^2^)	1.026 (0.911–1.154)	0.674		
CEA^a^ (per μg/L)	1.012 (0.996–1.029)	0.130		
ASA grade				
I				
II	2.086 (0.795–5.473)	2.086		
III	1.378 (0.256–7.406)	0.708		
Comorbidity				
Diabetes	1.378 (0.256–7.406)	0.708		
Chronic obstructive pulmonary disease	1.089 (0.192–6.187)	0.924		
Surgery				
Open resection (versus laparoscopic)	6.390 (1.900–21.491)	0.003*	6.798 (1.082–24.580)	**0.003**
Major (versus minor)	1.149 (0.549–2.402)	0.712		
Resection + RFA (versus only resection)	1.800 (0.658–4.920)	0.252		
Blood loss (per ml)	1.001 (1.000–1.001)	0.127		
Operation time (per min)	1.007 (1.00–1.015)	0.041*	1.009 (1.001–1.017)	**0.033**

*OR* odds ratio, *CI* confidence interval, *BMI* body mass index, *CEA* carcinoembryonic antigen, *ASA* American Society of Anesthesiologists, *RFA* radiofrequency ablation

Bold variables were considered statistically significant (*P* < 0.05). *Variables that were taken into the multivariable analysis (*P* < 0.010)

^a^28 patients missing

### Impact of surgery-related loss of muscle quantity and quality on survival

**Fig. 1:**
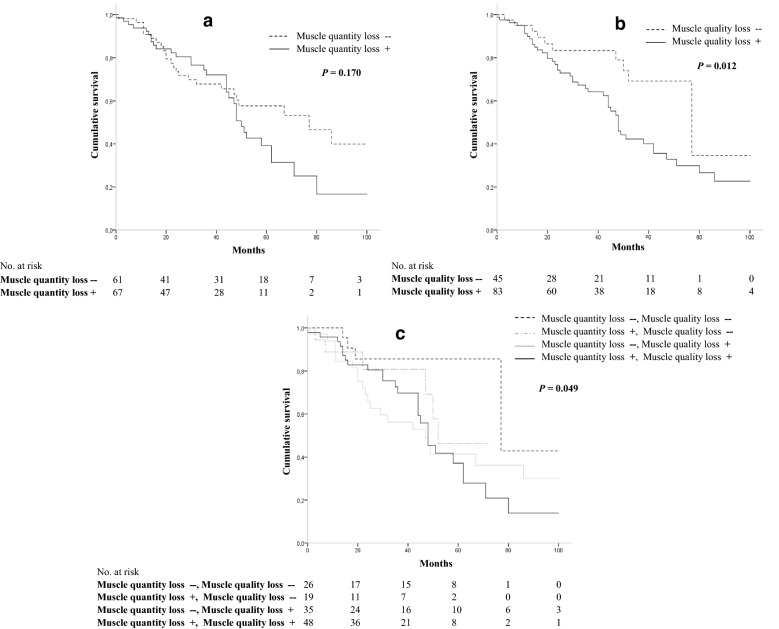
Impact of surgery-related muscle quantity and quality loss on survival in patients after liver resection for colorectal liver metastasis. **a** We found no significant difference in overall survival between patients with and without muscle quantity loss, and however, a trend was seen. **b** Patients with muscle quality loss had a significantly lower survival than patients without muscle quality loss (log-rank test, *P* = 0.012). **c** Patients without muscle quantity and quality muscle loss had significantly higher survival than other categories, while patients with both muscle quantity and quality loss had significantly lower survival (log-rank test, *P* = 0.049)

No significant differences in overall survival were found between patients with and without muscle quantity loss; however, a trend was seen (log-rank test, *P* = 0.170) (Fig. 1a). The overall survival in patients with muscle quality loss was significantly lower (log-rank test, *P* = 0.012) than that of patients without muscle quality loss (Fig. 1b). Patients without muscle quantity and quality muscle loss had significantly higher survival than other categories, while patients with both muscle quantity and quality loss had significantly lower survival (log-rank test, *P* = 0.049) (Fig. 1c).

## Discussion

The results of the present study demonstrated that more than half of our patients had surgery-related loss of muscle quantity (52%) and/or loss of muscle quality (65%). COPD and diabetes were risk factors for surgery-related loss of muscle quantity. A higher age, open resection and longer operation time were significantly associated with surgery-related muscle quality loss. The rate of postoperative complications was significantly higher in the group with surgery-related loss of muscle quality. Patients with both muscle quantity and quality loss had the lowest survival.

In accordance with the findings of Huang et al. [16] diabetes was an independent risk factor for loss of muscle quantity. Diabetes can have negative effects on skeletal muscle function [22]. The impaired insulin function may cause the loss of body protein, particularly during the katabolic postoperative period [23, 24]. In the current study, patients with COPD also had a greater risk at loss of muscle quantity. Studies have suggested that chronic obstructive COPD causes respiratory and limb muscle dysfunction [25, 26]. However, the mechanisms for how COPD contributes to abdominal muscle atrophy or for surgery-related loss of muscle quantity remain unclear [27, 28]. An underlying mechanism of why these patients were more prone to surgery-related muscle loss might be oxidative stress, which is inherent to pulmonary diseases such as COPD or asthma [29]. The oxidative stress could accelerate the process of muscle loss after surgery. However, due to the relatively small numbers of patients with COPD or diabetes, these results should be interpreted with caution and further research is needed to investigate the prognostic value of these two risk factors. Furthermore, a higher age was an independent predictor for surgery-related loss of muscle quality. Previous studies already found a correlation between a higher age and lower muscle quality [12] and quantity [16, 30, 31]. However, a correlation between a higher age and higher risk at surgery-related muscle loss has not been described before.

In this study, change in muscle quality was determined by the difference in AMA of the psoas on the pre- and postoperative CT-scan. All CTs were acquired after administration of intravenous contrast medium, according to the standard clinical protocol. This increases the radiodensity by 8 Hounsfield Units on average [32]. As all scans are acquired using the same contrast-enhanced protocol, the current results reflect the measurements in clinical setting and their direct usefulness in clinical practice. Low muscle radiation attenuation may be a reflection of increased water content (i.e., muscle edema) but is usually described as a marker for increased intramyocellular triglycerides (i.e., myosteatosis) [33, 34]. Though, in postoperative setting the decrease of muscle radiation attenuation should be interpreted with caution, since it might be influenced by perioperative fluid shifts. However, during the manual outlining of the muscle borders, the abdominal wall muscles in particular appeared to be affected by muscle edema and the presence of hematoma, especially in the operated right side, due to the subcostal incision, while the psoas muscles visually appeared unaffected by the direct surgical trauma and the surrounding fat did not show any edema. Therefore, in this study, it is assumed that the decrease in density is largely caused by myosteatosis and minimally caused by muscle edema.

Aoyama et al. [35] demonstrated that the greatest muscle loss occurs during the first postoperative week, and implied that this phenomenon was mainly because of increased catabolism caused by cytokine production under surgical stress. This mechanism is called the surgical stress response, which is the body’s response to prevent further injury through fluid conservation and substrate mobilisation. The surgical stress response comes with direct and indirect injury during surgery. Indirect surgical trauma occurs through events such as blood loss, alterations in blood pressure, and perfusion. Direct surgical injury is the result of incisions through different layers of the abdominal wall, the mobilisation of organs, and resection of organs or tissue [36]. The response starts with the release of cytokine and inflammatory mediators that control a complex process of metabolic, hormonal, and immunological processes, which subsequently results in the breakdown of muscle protein [37]. For example, surgical stress results in insulin resistance [38]. Myosteatosis has also been associated with insulin resistance, supporting the assumption that a postoperative decrease in muscle radiation attenuation of the psoas can be attributed to myosteatosis [34]. A higher degree of injury results in a higher peak and longer duration of cytokine release, as well as subsequent altered glucose metabolism, protein catabolism, and hormonal dysregulation [39]. Consequently, this leads to greater muscle loss. This explains why we found in our study that open surgery (versus laparoscopic) and a longer duration of the operation presented to be risk factors for surgery-related loss of muscle quality. This knowledge highlights the importance of minimally invasive surgery.

To our knowledge, there is only one previous study that investigated surgery-related changes in muscle quality analysed with abdominal CT-scan on outcome, rather than only muscle quantity [18]. Kobayashi et al. [18] investigated postoperative changes in skeletal muscle mass and muscle quality, and presented that postoperative loss of skeletal muscle quality was an independent risk factor for the recurrence of hepatocellular carcinoma in patients following a hepatectomy for hepatocellular carcinoma. Our study demonstrated that patients without loss of muscle quantity and quality had significantly higher survival than other categories, while patients with both loss of muscle quantity and quality had significantly lower survival. Moreover, in our study the rate of postoperative complications was significantly higher in the group with surgery-related loss of muscle quality. These results emphasizing the need for further investigation into the aetiology and occurrence of surgery-related muscle loss and possible pathways for prevention [40]. Despite it has previous described that postoperative complications rates were higher in patients with surgery-related muscle quantity loss, [16] it is not yet clear whether the significant higher rates of postoperative complications were a cause or effect of surgery-related muscle quantity or quality loss. However, in the light of this: in contrast to other studies, postoperative CT scans in most patients were routinely performed as a standard protocol [9]. In this manner, we prevented the bias that CT scans probably create, because they are performed during a complicated course, which could theoretically influence the percentage of patients with SML.

Through identifying risk factors for SML, a perioperative intervention may prevent or reduce SML and subsequently improve postoperative outcomes. Two important risk factors that contributes to muscle loss are malnutrition and inactivity. After surgery, adequate protein supplementation (1.5 g of protein per kg of body weight/day) can influence the surgical stress response and postoperative catabolism and subsequently treat declines in muscle mass, muscle strength, and functional capacity [41]. Also sufficient physical activity is critical for preventing muscle loss in patients [41, 42]. A combination of adequate protein intake and sufficient exercise has been shown to facilitate muscle gain [43–45]. Because of the retrospective nature of our study, data on postoperative protein and specific data on the degree of mobilisation of the included patients in our study were lacking. This lack of data are limitations of our research. Further research into postoperative protein intake and physical activity will be essential for setting up a proper and feasible protocol to prevent patients suffering muscle loss [37, 40]. Furthermore, a promising idea might be the application of neuromuscular electrical stimulation to muscles to maintain muscle thickness after surgery [46]. In conclusion, the current study demonstrated that surgery-related loss of muscle quantity or quality is present in more than half of the patients after liver resection for CRLM. Specific risk factors for SML could be identified. Overall survival was lowest in patients with both muscle quantity and quality loss, showing that surgery-related loss of muscle quality and quantity may be used in predicting prognosis. To reduce muscle loss, a perioperative programme focused on adequate protein intake combined with early mobilisation, especially for patients in risk groups, could be the first step.

## Data Availability

Data are available from the corresponding author on reasonable request.
